# A Partial Structural and Functional Rescue of a Retinitis Pigmentosa Model with Compacted DNA Nanoparticles

**DOI:** 10.1371/journal.pone.0005290

**Published:** 2009-04-24

**Authors:** Xue Cai, Zack Nash, Shannon M. Conley, Steven J. Fliesler, Mark J. Cooper, Muna I. Naash

**Affiliations:** 1 Department of Cell Biology, University of Oklahoma Health Sciences Center, Oklahoma City, Oklahoma, United States of America; 2 Departments of Ophthalmology and Pharmacological & Physiological Science, Saint Louis University School of Medicine, St. Louis, Missouri, United States of America; 3 Copernicus Therapeutics, Inc., Cleveland, Ohio, United States of America; University of Florida, United States of America

## Abstract

Previously we have shown that compacted DNA nanoparticles can drive high levels of transgene expression after subretinal injection in the mouse eye. Here we delivered compacted DNA nanoparticles containing a therapeutic gene to the retinas of a mouse model of retinitis pigmentosa. Nanoparticles containing the wild-type retinal degeneration slow (*Rds*) gene were injected into the subretinal space of *rds^+/−^* mice on postnatal day 5. Gene expression was sustained for up to four months at levels up to four times higher than in controls injected with saline or naked DNA. The nanoparticles were taken up into virtually all photoreceptors and mediated significant structural and biochemical rescue of the disease without histological or functional evidence of toxicity. Electroretinogram recordings showed that nanoparticle-mediated gene transfer restored cone function to a near-normal level in contrast to transfer of naked plasmid DNA. Rod function was also improved. These findings demonstrate that compacted DNA nanoparticles represent a viable option for development of gene-based interventions for ocular diseases and obviate major barriers commonly encountered with non-viral based therapies.

## Introduction

Gene therapy represents, in theory, the ultimate desirable strategy for long-term treatment of inherited retinal diseases. Viral delivery of exogenous genes has been used successfully in the eye for the treatment of inherited blinding diseases in mice and dogs [1]–[4]. Non-viral delivery methods represent an additional therapeutic strategy, but historically these approaches have been limited by inefficient entrance of the genetic material into the target cells and by attenuated duration of transgene expression [5], [6]. In the present study, we have adopted a non-viral gene transfer strategy designed to overcome these barriers. Our approach is to use single-molecule DNA nanoparticles in which plasmid DNA is compacted by polyethylene glycol (PEG)-substituted 30-mer lysine peptides (CK30PEG). These particles have several advantages over traditional non-viral approaches: they are versatile, small in size, easy to prepare, have large vector capacity (up to 20 kb), are stable in nuclease-rich environments, and have high transfectivity [7]–[11]. Their high transfectivity is due, in part, to the small particle size (diameter<8 nm [9], [10]) and also to specific interactions with cell-surface nucleolin and subsequent non-degradative trafficking to the nucleus [12]. These nanoparticles can successfully transfect both dividing and non-dividing cells, and have been shown to be effective agents, both in experimental models as well as in a phase I/IIa clinical trial for cystic fibrosis, in delivering genes of interest to multiple tissues, including the lung, retina, and brain [7], [8], [10], [11], [13]–[15]. Such nanoparticles are non-inflammatory, non-immunogenic [7], [14], [16], and our own proof-of-principle studies have demonstrated that they are non-toxic in the eye [13].

Recently, we showed that CK30PEG nanoparticles containing a CMV-EGFP plasmid could be used to safely and efficiently transfer genes to the eyes of adult wild-type (WT) mice [13]. The high ocular transfectivity of the particles is evident in our observation that nearly all retinal photoreceptor cells were transfected (in multiple regions examined), not just those cells near the site of injection. Furthermore, gene expression levels could be titrated to mimic the expression levels of native photoreceptor genes. Based on our initial results and those of others, this strategy appears to be a good candidate for the delivery of therapeutic genes to rescue hereditary ocular diseases.

We chose to use a murine model of retinitis pigmentosa (the *rds^+/−^* mouse) for our first disease rescue studies. The protein product of this gene, RDS (retinal degeneration slow; also called Peripherin/rds or Peripherin 2), is a tetraspanin glycoprotein known to form homomeric complexes as well as heteromeric complexes with a related tetraspanin protein, rod outer segment membrane protein 1 (ROM-1). RDS is photoreceptor-specific and is critical for photoreceptor disc rim assembly, outer segment (OS) orientation, photoreceptor structural stability, and OS disc renewal [17]–[19]. Over 80 different mutations in the *RDS* gene have been identified in humans and are associated with multiple retinal diseases, including autosomal dominant retinitis pigmentosa (adRP) and progressive macular degeneration (MD) [20]–[23]. Unlike the retina in the homozygous (*rds* ^−/−^) mouse, which fails to form OSs and undergoes fairly rapid apoptotic photoreceptor cell death, the retina in the heterozygous (*rds^+/−^*) mouse exhibits a classic, well-defined adRP phenotype characterized by early onset rod degeneration and late onset cone degeneration. Furthermore, the OSs of the *rds^+/−^* are highly disordered, malformed, and short (compared to normal OSs), are electrophysiologically deficient, and express reduced levels of key phototransduction proteins [24]–[27]. Of relevance to our strategy of gene supplementation, we and others have shown that expression of at least 80% of the normal amount of RDS is necessary in order to build proper photoreceptor OSs [24], [28].

The purpose of the current study was to test the efficacy of CK30PEG nanoparticles with regard to their ability to rescue the *rds^+/−^* phenotype as a prelude to optimization of this technology for the treatment of human hereditary eye diseases. It is known that the *rds* model is challenging to rescue, because of the severe structural defect associated with the complete absence of RDS protein [26], and few groups are using it as a gene therapy model in spite of the multitude of *RDS*-associated diseases. One other group has documented partial rescue of an *rds* model with gene transfer therapy using an AAV vector [29]–[31], and we have shown that the disease phenotype can be rescued by transgenesis [24]. Here we present results showing that compacted DNA-nanoparticles containing the *Rds* gene are capable of achieving significant rescue of the disease phenotype in the *rds* ^+/−^ adRP model.

## Results

### *Rds* nanoparticles drive high and persistent transgene expression

RDS expression and localization to the distal connecting cilium in the mouse rod photoreceptor cell begin around postnatal day 5 (P5) [26], [32] (*i.e.*, before OS formation), a time that precedes the onset of retinal degeneration in the *rds* model. Hence, we selected P5 as the physiologically appropriate developmental stage for therapeutic intervention. Two vectors were generated, each expressing the full-length cDNA of normal mouse peripherin/rds (NMP), one under the control of the ubiquitously expressed chicken beta-actin promoter (CBA), and the other employing the well characterized photoreceptor-specific promoter for the human interphotoreceptor retinoid-binding protein (IRBP) [33]. Acetate compacted nanoparticles containing the vectors (Figure S1) or controls were injected subretinally into *rds^+/−^* mice at P5 and followed for up to four months. The controls chosen for this study were saline (vehicle) and uncompacted plasmid DNA (called “naked DNA”) carrying the same therapeutic transgene (CBA-NMP or IRBP-NMP).

**Figure 1:**
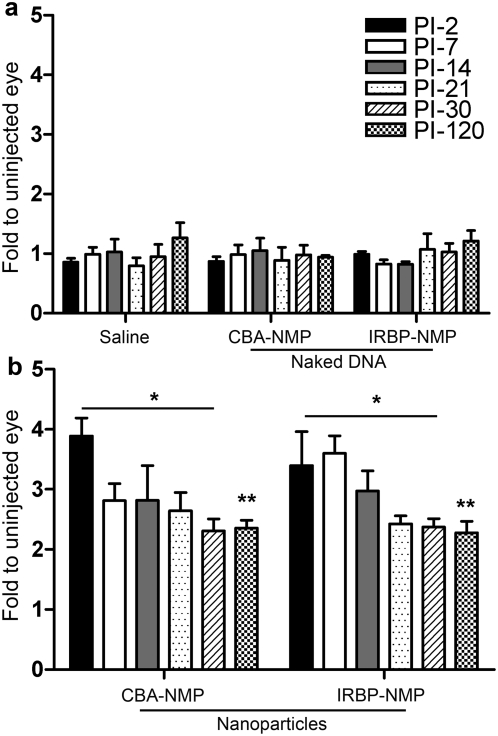
Injection of NMP nanoparticles into P5 *rds* ^+/−^ animals increases *Rds* mRNA levels. cDNA from eyes injected with saline, naked DNA (A) or nanoparticle DNA (B) at PI-2 through PI-120 was prepared and analyzed by qRT-PCR to determine relative *Rds* mRNA levels. Because *Rds* primers amplify from the NMP (nanoparticle) and the WT (endogenous) allele but not from the *Rds* mutant allele, expression values are reported as fold change from the uninjected contralateral control eye. Values shown are averages±S.D. (N = 3–6 mice per group). (A) Injection of saline or naked DNA does not alter *Rds* message levels at any time point. (B) Conversely, injection of both CBA-NMP and IRBP-NMP compacted DNA nanoparticles leads to a significant, two- to four-fold increase in total *Rds* message level compared to the naked DNA injected eyes (*p<0.001, **p<0.05. This increase persists through the last time point examined (PI-120).

As shown in Figure 1, injection of both CBA-NMP and IRBP-NMP nanoparticles resulted in significantly elevated expression of *Rds* message, as measured by qRT-PCR. At post-injection day 2 (PI-2), mRNA levels in CBA-NMP and IRBP-NMP nanoparticle- injected eyes were at least three- to four-fold higher than the saline or naked DNA-injected eyes (Figure 1). Eyes injected with IRBP-NMP maintained elevated expression until PI-14, then stabilized at levels two- to three-fold higher than controls, while eyes injected with CBA-NMP stabilized at similar levels at PI-7. Neither saline nor naked DNA produced a significant alteration in *Rds* mRNA levels (Figure 1A), compared to uninjected eyes. Elevated mRNA levels were maintained for up to four months (PI-120), the longest time point examined.

### Compacted DNA nanoparticles efficiently transfer RDS to all photoreceptor cells

**Figure 2:**
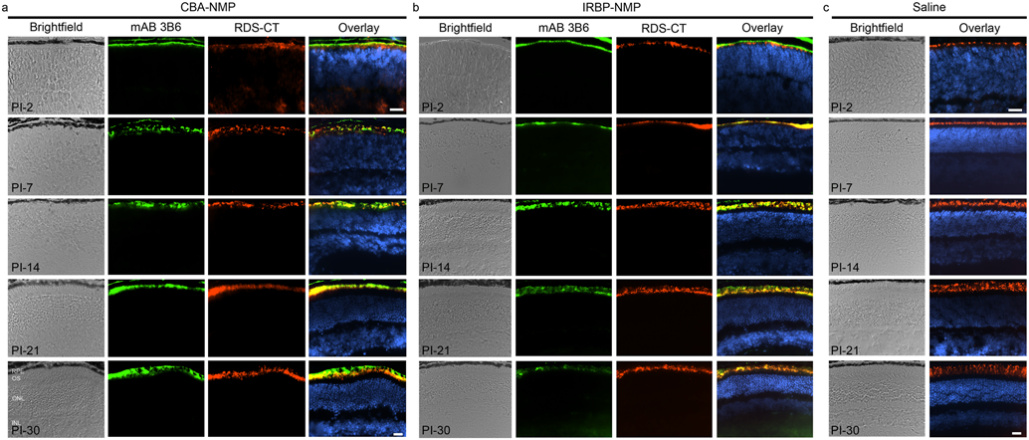
Transferred NMP co-localizes with endogenous RDS. Frozen retinal sections from eyes collected at multiple ages (PI-2 to PI-30) were immunostained for NMP (mAB 3B6, green) and total RDS (RDS-CT, red) with a nuclear counterstain (DAPI, blue). Transferred RDS from eyes injected with CBA-NMP (A) and IRBP-NMP (B) nanoparticles is detected at PI-2. Expression remains strong through the latest time point analyzed (PI-30) and co-localizes with native RDS. Expression is limited to the OSs or nascent OSs and is not detected in any other retinal cell types, subcellular compartments or layers. (C) No NMP is detected in saline-injected control eyes, but native RDS is detected beginning at PI-2 (P7), consistent with normal ocular development. Scale bars, 20 µm. N = 3–5 mice per group. Abbreviations: RPE, retinal pigment epithelium; OS, outer segment layer; ONL, outer nuclear layer; INL, inner nuclear layer.

We next examined the identity of the cells that took up the exogenously delivered NMP cDNA and the efficiency of gene product expression within the retina over time, using immunohistochemistry. The entire eye was cut and every sixth section was collected and assessed, enabling us to examine gene expression in multiple regions throughout the retina. Due to an epitopic modification in the NMP carboxyl terminus (P341Q), which is not present in wildtype RDS and does not result in retinal disease or vision loss [24], the transferred *Rds* gene product can be detected selectively even on a normal RDS background using a monoclonal antibody (mAB 3B6) that recognizes the P341Q epitope. [For demonstration of the selectivity of mAB 3B6 for transferred RDS (NMP) as opposed to native (endogenous RDS), see Figure S2 and our previous publications [24], [34].] The endogenous mouse RDS protein (and to a much lesser extent transgenic NMP protein) is labeled with the RDS-CT antibody [35]. Although normal OS development has not yet begun at P7 (PI-2) [36], Figure 2 (top row) shows expression of both transferred (Figure 2A and 2B) and native RDS (Figure 2C) protein in the tip of the photoreceptors. By PI-7 (P12), distinct outer and inner nuclear layers are apparent and NMP/RDS staining in the tips of nascent OSs is visible as a thin immunopositive layer adjacent to the photoreceptor nuclei. NMP distribution in the OSs persisted through the latest time point examined (PI-30). NMP also co-localized with native RDS and was usually limited to the OS layer (Figure 2); no NMP was detected in eyes injected with saline (Figure 2C and Figure S3) or naked DNA (not shown). Occasionally, NMP expression was detected in RPE cells after nanoparticle injection (Figure 2). Expression in the RPE was highly variable; while many animals had some RPE expression, others did not. RPE expression was much more common in eyes injected with CBA-NMP nanoparticles, consistent with its role as a ubiquitous promoter. On the other hand, IRBP-NMP staining in the RPE was almost always limited to PI-2. NMP detection with mAB 3B6 in the OSs was heterogeneous, with stronger signal in areas closer to the injection sites. However, we estimate that a majority of photoreceptors expressed the product of the transferred gene (based on a qualitative assessment of mAB 3B6 immunostaining in successive retinal sections). The choice of promoter did not have any apparent effect on cellular distribution within the photoreceptor: both CBA-NMP and IRBP-NMP nanoparticles exhibited similar distribution patterns at all time points examined.

### *Rds* nanoparticles improve expression levels of key visual transduction proteins

**Figure 3:**
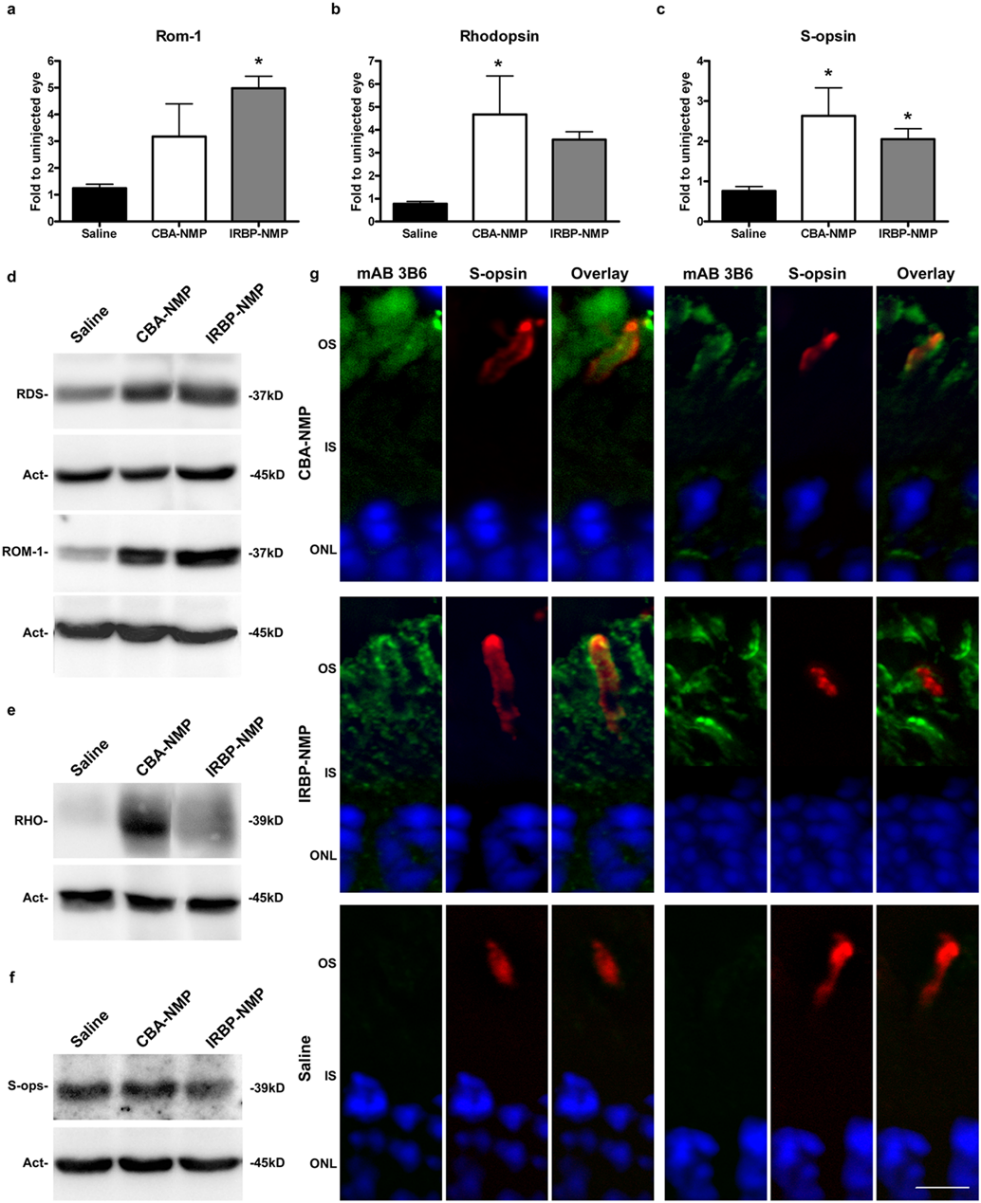
Transferred NMP leads to increased expression of photoreceptor-specific proteins in the *rds* ^+/−^ retina. (A–C) cDNA was collected at PI-30, and message levels of photoreceptor genes were analyzed by qRT-PCR. (A) CBA-NMP nanoparticle injection leads to a modest increase in *Rom-1* message levels, while IRBP-NMP nanoparticle injection increases expression four- to five-fold over levels in uninjected control eyes. (B,C) CBA-NMP and IRBP-NMP nanoparticle injections lead to increases in rod (B) and cone (C) opsins. (A–C); N = 3 animals per group. (D–F) Protein levels at PI-30 after nanoparticle injection were examined. Representative SDS-PAGE/Western blots from individual retinas are shown (N = 5–6 animals per group). (D) CBA-NMP and IRBP-NMP nanoparticle injections increase RDS and ROM-1 protein levels (protein load: 20 µg per lane). (E) Increases in rhodopsin protein (RHO) are detected after injection of both CBA-NMP and IRBP-NMP nanoparticles (protein load: 10 µg per lane). (F) No change in S-opsin (S-ops) protein level is detected after nanoparticle injection (protein load: 50 µg per lane). (G) Double immunolabeling for transferred RDS (mAB 3B6, green) and cone OSs (S-opsin, red) with nuclear counterstain (DAPI, blue) was performed on frozen sections from PI-30 eyes. Representative cones from two different animals are shown for each treatment. Cones in eyes injected with CBA-NMP or IRBP-NMP nanoparticles express transferred NMP (top and middle rows). Saline injected eyes express no transferred NMP (bottom row). Scale bar, 5 µm; N = 3–5 animals per treatment group. Abbreviations: OS, outer segment layer; IS, inner segment layer; ONL, outer nuclear layer * = p<0.05.

Our next step was to determine whether nanoparticle-driven expression of NMP results in rescue of the *rds* ^+/−^ disease phenotype. To measure biochemical rescue, we assayed the levels of several photoreceptor-specific proteins known to be decreased by RDS deficiency. Figure 3 (panels A and D) shows that expression levels of the RDS binding partner ROM-1 were increased, both in terms of message (by qRT-PCR) and protein (by Western blot analysis), compared to uninjected controls, at PI-30. Consistent with the mRNA data presented in Figure 1, expression of RDS protein was also increased in NMP nanoparticle-injected eyes. Expression of rhodopsin (the rod visual pigment) is necessary for phototransduction and proper photoreceptor maintenance, and is significantly decreased in the *rds* ^+/−^ retina [37]. We show that injection of NMP nanoparticles led to increased rhodopsin message (Figure 3B) and protein (Figure 3E) levels. We also observed a similar increase in the message level of short-wavelength cone opsin (S-opsin, Figure 3C) after nanoparticle injection, although no alteration in S-opsin protein level was detected (Figure 3F), likely due to lack of cone degeneration at this age.

Since the photoreceptor population in the mouse retina consists of 95–97% rods [38], [39], the results presented in Figure 2 are consistent with the conclusion that the two types of nanoparticles drove gene expression in rods and that their products were delivered with fidelity to the OS. However, it was not clear from those data whether transferred RDS protein was expressed in cones. Therefore, double labeling for NMP and S-opsin was performed on PI-30 eyes. Two representative cones from each nanoparticle-injected and control eye are shown in Figure 3G (single, 0.5-µm slices of spinning disk confocal image stacks). Most cones from nanoparticle-injected eyes expressed NMP when consective sections were evaluated from the same eye. S-opsin immunopositive cone cells that lacked NMP expression were mainly located in areas far from the injection site. No NMP-positive cells were detected in saline-injected eyes (Figure 3G, bottom row).

### Nanoparticle-driven *Rds* expression restores retinal function

The *rds* ^+/−^ mouse adRP model exhibits reduced electroretinogram (ERG) responses indicative of early-onset slow rod degeneration followed by late-onset slow cone degeneration [26], [40]. In order to assess functional rescue of this phenotype after treatment, full-field ERGs were obtained from nanoparticle-injected and control mice. Initial ERGs were obtained and analyzed at PI-30 (see Table 1). Average scotopic a-wave amplitudes, indicative of rod function, were increased with statistical significance after injection of either CBA-NMP or IRBP-NMP nanoparticles, compared to amplitudes from eyes injected with naked DNA or saline. In order to confirm that the naked DNA had no adverse effect, a subset of animals was injected with saline only. Scotopic a-wave amplitudes for saline injected animals were not significantly different from those injected with either CBA-NMP or IRBP-NMP naked DNA (p = 0.2634). Interestingly, nanoparticles led to an improvement in cone function. The magnitude of rescue varied considerably with both nanoparticles, most likely due to variations in particle uptake and/or relative activity of CBA *vs*. IRBP promoters in rods and cones. Several nanoparticle-injected animals exhibited significantly greater-than-average rescue; 6/15 (IRBP-NMP) and 5/19 (CBA-NMP) treated animals had 90% increase in scotopic a-wave amplitudes, compared to naked DNA-injected controls. Similarly, 4/15 (IRBP-NMP) and 6/19 (CBA-NMP) animals had at least a 70% increase in cone ERG amplitudes.

**Table 1:** Average full-field ERG values at various timepoints.

		Nanoparticle		Naked DNA		Change^b^	P^b^
		Average^a^±SEM	#^c^	Average^a^±SEM	#^c^		
**PI-30**							
**Scotopic-A**	**CBA-NMP**	134.8±13.3	19	92.9±9.4	6	41.9 µV, 45.1%	0.018
	**IRBP-NMP**	146.7±13.7	15	96.0±11.0	9	50.7 µV, 52.8%	0.018
**Photopic-B**	**CBA-NMP**	148.1±11.3	19	98.7±15.2	6	49.4 µV, 50.1%	0.035
	**IRBP-NMP**	134.4±13.7	15	92.4±9.7	9	42.0 µV, 45.4%	0.040
**PI-60**							
**Scotopic-A**	**CBA-NMP**	107.7±7.8	5	81.8±17.2	4	25.9 µV, 31.7%	0.061
	**IRBP-NMP**	148.4±8.9	10	70.5±18.8	4	77.9 µV, 110.4%	0.011
**Photopic-B**	**CBA-NMP**	117.3±19.7	5	131.0±11.3	4	−13.7 µV, −11.5%	0.594
	**IRBP-NMP**	194.8±16.0	10	64.5±20.4	4	130.3 µV, 202.0%	0.0007
**PI-120**							
**Scotopic-A**	**CBA-NMP**	123.9±12.9	5	77.1±17.3	6	46.8 µV, 60.7%	0.086
	**IRBP-NMP**	129.4±9.6	5	67.6±16.2	5	61.8 µV, 91.4%	0.011
**Photopic-B**	**CBA-NMP**	120.8±14.83	5	108.0±13.48	6	12.8 µV, 11.8%	0.54
	**IRBP-NMP**	185.6±20.6	5	87.9±20.5	5	97.7 µV, 111.1%	0.009

a Values are mean µV±S.E.M.

b Comparison between nanoparticle and naked DNA using 2-tailed un-paired Student's t-test as described in methods.

c Number of animals tested.

Although WT eyes can completely recover from P5 subretinal injections, we observed that ERG amplitudes from saline- and naked DNA-injected eyes in the *rds^+/−^* tended to be lower than in uninjected eyes (data not shown). These data, in combination with our earlier work on adult *rds* ^+/−^ mice [41], suggest that the *rds* ^+/−^ eye is more fragile than the normal eye and that subretinal injections *per se* in the mutant may cause adverse effects on visual function which would need to be overcome by any treatment. This idea is further supported by the wide variation in nanoparticle-mediated functional rescue, and highlights the need to assess rescue in every treated animal.

**Figure 4:**
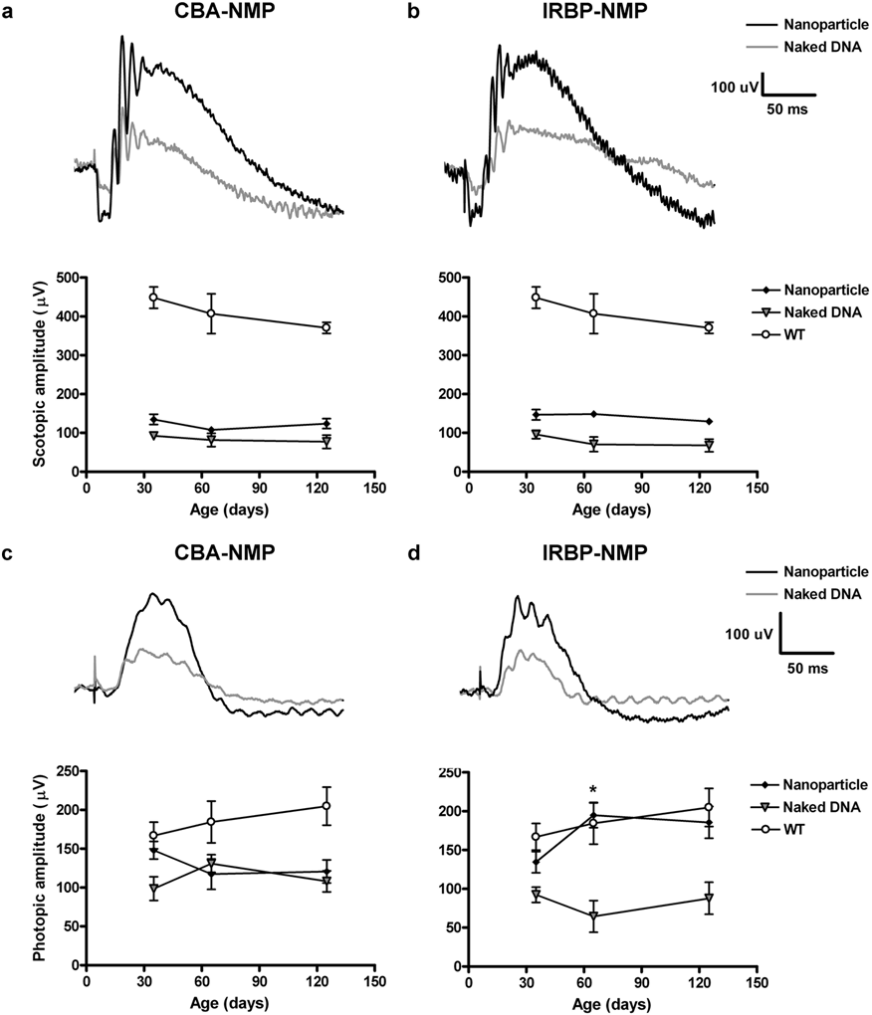
Expression of transferred NMP leads to partial functional rescue of the *rds* ^+/−^phenotype. A subset of individual injected animals identified from PI-30 ERG analysis (Table 1) was chosen for follow-up. (A,B) Top: scotopic traces from naked DNA (*gray*) and nanoparticle (*black*) injected eyes at PI-30. Bottom: (A) Scotopic a-wave amplitudes from eyes injected with CBA-NMP nanoparticles are elevated at PI-30, but drop almost back to baseline at PI-60 and PI-120. (B) IRBP-NMP nanoparticle-injected animals retain improved rod function (as measured by scotopic a-wave) through PI-120. (C,D) Top: photopic traces from naked DNA (*gray*) and nanoparticle (*black*) injected eyes at PI-30. Bottom: (C) Cone function (as measured by photopic b-wave) does not remain substantially improved past PI-30 in eyes injected with CBA-NMP nanoparticles. (D) Photopic b-wave amplitudes in IRBP-NMP nanoparticle-injected animals are improved at PI-30 and continue improving at PI-60 (* = p<0.05, PI-30 vs. PI-60) before stabilizing at the last time point examined (PI-120). Amplitudes are means±standard error (N values are in Table 1).

In order to determine whether functional rescue persisted at later timepoints, animals that demonstrated the hallmarks of rescue at PI-30 were selected for follow-up at PI-60 and PI-120 (Table 1 and Figure 4). Injection of CBA-NMP nanoparticles did not result in long-term functional rescue of rods (Table 1, and Figure 4A, bottom) or cones (Table 1, and Figure 4C, bottom). In striking contrast, ERG amplitudes from IRBP-NMP nanoparticle-injected eyes continued to be elevated at both PI-60 and PI-120 when compared to naked DNA-injected controls (Table 1, and Figure 4B and 4D, bottom). Cone function continued improving between PI-30 and PI-60 (p<0.01) before stabilizing near WT levels at PI-120; based on two-way ANOVA, age was not an interacting factor in any case. In addition to the overall (average) improvement in cone ERG function at PI-120, in 6/10 (PI-60) and 2/5 (PI-120) cases IRBP-NMP nanoparticle injection led to photopic ERG levels that exceeded the mean value for uninjected WT animals (for example, at PI-120, treated subject 1, 245.6 µV *vs*. age-matched WT average 204.9±24.5 µv, N = 8). This suggests that IRBP-NMP nanoparticle-mediated NMP expression is capable of overcoming damage due to subretinal injection and can slow or rescue the functional degeneration associated with RDS haploinsufficiency.

### OS ultrastructure is substantially improved by increased *Rds* expression

**Figure 5:**
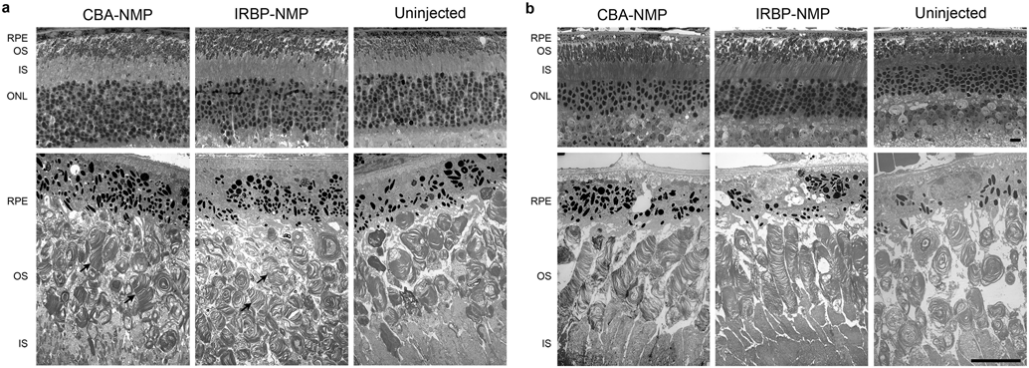
Transferred NMP leads to structural rescue of the *rds* ^+/−^ phenotype. Light micrographs (top row) and electron micrographs (bottom row, N = 3–5 animals per group) from *rds* ^+/−^ were examined. (A) At PI-30, moderate ultrastructural rescue is detected in the OSs of nanoparticle injected eyes (arrows). (B) By PI-120 significant ultrastructural improvement in OSs of nanoparticle injected eyes is apparent. OS discs are properly aligned and flattened and OS do not exhibit the swirl-like structures typical of the *rds* ^+/−^. RPE, retinal pigment epithelium; OS, outer segment layer; IS, inner segment layer; ONL, outer nuclear layer. Scale bar, 10 µm.

Finally, we analyzed NMP nanoparticle-mediated structural rescue of photoreceptors in the *rds* ^+/−^ retina, using both light and electron microscopy, at PI-30 and PI-120, in comparison with uninjected controls. Photoreceptors in the *rds* ^+/−^ retina typically exhibit very short OSs with misaligned and whorl-like disc membranes. At PI-30, there was a modest increase in outer nuclear layer (ONL) thickness (Fig 5A, top), and many individual OSs exhibit improved ultrastructure (arrows, Figure 5A, bottom). By PI-120, however, virtually all photoreceptors examined showed noticeable structural improvement (Figure 5B). Consistent with the ERG results (see Figure 4), structural rescue was more pronounced in the IRBP-NMP-injected eyes compared to CBA-NMP-injected eyes at PI-120, but both exhibited OSs with orderly stacks of disc membranes.

In order to determine the extent of these structural improvements, we undertook a series of morphometric analyses of the nanoparticle injected-eyes compared to controls. Histological images were collected from each eye at 200 µm, 400 µm, and 600 µm from the optic nerve head (both temporally and nasally) and vertical rows of ONL nuclei and OS thickness were measured. Figure S4 shows results from two representative experimental animals, with the average values (± standard deviation) obtained from uninjected control eyes shaded in gray (accompanying supplemental methods found in Text S1). At PI-30, injected animals showed little or no increase in the number of rows in the ONL (top panels), but a definite increase in OS thickness (bottom panels). At PI-120, there was no morphometric evidence of any histological benefit from the CBA-NMP nanoparticles, while IRBP-NMP nanoparticles led to a slight increase in both the number of rows in the ONL and in OS thickness. The increase in ONL rows at PI-120 is particularly relevant, since it suggests that nanoparticle-mediated increases in RDS may slow photoreceptor cell death in the *rds* ^+/−^.

## Discussion

This study demonstrates significant rescue of an ocular disease phenotype with a non-viral gene delivery method. Our data indicate that ocular delivery of compacted DNA-nanoparticles carrying *Rds* cDNA at P5 results in transgene expression that is: a) rapid-onset (starts at PI-2) and high with levels elevated up to four-fold above the endogenous, b) widely distributed in all photoreceptors, c) properly localized to the OSs of rods and cones, and d) persistently detected up to 4 months post- treatment without any obvious adverse side effects. Nanoparticle injection also improved expression of key photoreceptor-specific proteins known to be affected by the ongoing photoreceptor degeneration in the *rds* ^+/−^ retina. Notably, IRBP-NMP nanoparticles afforded significant and persistent restoration of both rod and cone function, with full-field cone ERG amplitudes approaching those seen in WT mice. Ultrastructural rescue in nanoparticle-injected eyes was similarly pronounced; at four months post- treatment, IRBP-NMP animals exhibited properly oriented OSs with well-aligned discs.

Viral gene therapy has been remarkably successful in treating some types of ocular diseases, the most notable example of which to date being the AAV-mediated long-term rescue of vision in Briard dogs harboring a mutation in RPE65 [1], [2]. Results from these studies have prompted three successful and ongoing human clinical trials [42]–[44]. However, the development of effective non-viral vectors is a prudent step as an alternative to more traditional methods. While a number of non-viral approaches have been explored, including the use of liposomes, electroporation of naked DNA, and gene delivery with dendrimers, they have encountered persistent problems with limited uptake and short-term gene expression [6], although advances in this area have been made [45]. Therefore, we chose to test the efficacy of compacted DNA nanoparticles comprised of PEG-substituted lysine peptides for gene delivery. We show here that these nanoparticles represent a significant improvement over other non-viral vectors in terms of transgene uptake and stability of expression (elevated levels detected to the latest timepoint examined PI-120). Furthermore, we show partial morphological and functional rescue of the *rds* ^+/−^ model. Because of the structural defects that accompany *Rds* mutations or deficiency, rescue of the disease phenotype heretofore has been particularly difficult [29]–[31]. However, since most *Rds*-associated RP in humans is due to loss-of-function mutations causing a haploinsufficiency phenotype, the *rds^+/−^* is a relevant model to target.

Since the murine *Rds* promoter has not been fully characterized, in this study we chose two other well-studied promoters to drive transgene expression: one is a ubiquitously expressed promoter (CBA), while the other is tissue-specific (IRBP). One of our goals was to see which of these two promoters would provide the best transfectivity and rescue. Based on previous studies using these promoters in the eye, we initially hypothesized that the IRBP promoter would drive modest expression selectively in rods and cones [33], while the CBA promoter would direct high expression levels in multiple ocular cell types. Although the CBA promoter has been shown to drive GFP expression in most ocular tissues soon after injection, it has also been documented to dictate a more restricted distribution of tissue-specific genes in the eye [46]. Our study confirmed this latter point; the product of CBA-NMP-driven transgene expression was almost exclusively detected in the photoreceptor OSs, with occasional expression in the RPE. This more specific tissue distribution is likely due to the rapid turnover of any ectopically expressed protein.

IRBP-mediated NMP expression was detected in rods and cones, but immunohistochemistry revealed that while most cones expressed the transferred gene, some did not. The reason for this variation is not known, but it is possible that cones differentially express the nucleolin cell-surface protein known to mediate uptake of the nanoparticles [12]; alternatively, heterogeneity in the response to the injection insult may affect NMP expression/uptake. In spite of variation in nanoparticle-driven gene expression in cones, we observed essentially complete functional cone recovery (to WT levels) in many IRBP-NMP treated animals. Cone rescue is likely of greater magnitude than rod rescue because cones need less RDS to form fully functional OS than do rods [24]. Furthermore, the RDS haploinsufficiency phenotype is much less severe in cones than in rods, and we have recently demonstrated a differential role for RDS in the two photoreceptor types [47].

Due to the more pronounced functional and structural rescue in eyes treated with IRBP-NMP nanoparticles (compared to CBA-NMP), we conclude that IRBP is the preferred promoter for this application and will take advantage of this finding in future studies directed at therapeutic intervention in hereditary photoreceptor degenerations. It is not clear to us why IRBP-NMP particles lead to more pronounced cone rescue than do CBA-NMP particles in spite of demonstrating similar levels of mRNA expression (Figure 1), but it is possible that the two transcripts are differently regulated. While the IRBP promoter drives gene expression in both rods and cones, qualitative results from transgenic mice made with the same transgene as the IRBP-NMP nanoparticles (Figure S5 and [24]) suggest that IRBP-mediated gene expression is much higher in cones than in rods. The enhanced cone rescue in IRBP-NMP (compared to CBA-NMP) treated eyes is likely a result of this inherent ability of the IRBP promoter to drive higher transgene expression in cones.

We chose to treat our animals at P5 both because it represents a physiologically appropriate intervention time and because previous *rds* gene therapy trials have reported difficulties correcting the ERG defect in adult *rds* mice, whereas correction was observed following neonatal gene transfer [30], [31]. The high rates of transfectivity we report here after P5 injection of *rds* ^+/−^ mice combined with the high rates of transfectivity we previously reported after subretinal injection in adult WT mice [13] show that these DNA-nanoparticles can effectively transfect both mitotic and post-mitotic (terminally differentiated) retinal cells. Furthermore, our ability to drive long-term expression (up to four months) suggests that CK30PEG-compacted DNA-nanoparticles may not be subject to some of the practical impediments that have limited the utility of other forms of non-viral gene therapy. We have shown partial structural and functional rescue of the *rds* ^+/−^ phenotype by delivery of compacted DNA-nanoparticles containing WT *Rds*. These nanoparticles offer a promising gene therapy modality that, with further development, may find practical applications in the treatment of a wide variety of hereditary ocular diseases.

## Materials and Methods

### Ethics statement

All animal procedures were approved by the University of Oklahoma Health Science Center Institutional Animal Care and Use Committee (IACUC) and adhered to the ARVO Statement for the Use of Animals in Ophthalmic and Vision Research (see Policies, http://www.arvo.org/).

### Vector and nanoparticle construction

Two constructs were generated expressing the full-length mouse *Rds* cDNA (1.7 kb) containing the P341Q modification (called NMP), for specific detection with the mAB 3B6 monoclonal antibody [18]. Either the human interphotoreceptor retinoid-binding protein (IRBP) promoter (1.3 kb) [33] or the chicken beta-actin (CBA) promoter (280 bp) was used to drive NMP expression. The two promoter regions were amplified from genomic DNA by PCR, sequenced, and then sub-cloned into the pXL-TOPO vector in front of NMP using EcoR I and BamH I restriction enzymes. The two plasmid DNAs were individually compacted into rod-like acetate nanoparticles (Figure S1) at Copernicus Therapeutics as reported previously [12], [13], and were used at a final concentration of 3.06 µg/µl in 0.9% saline.

### Subretinal injections

Mice (*rds* ^+/−^ pups at P5) were anesthetized by incubation on ice for 2–2.5 minutes. The eyelid of the right eye was cut, the cornea was exposed, and a puncture in the cornea was made with a sterile 30-gauge needle. A 35-gauge blunt-end needle attached to a 10 µl Nanofil® syringe (World Precision Instruments, Sarasota FL) was inserted into the puncture under an operating microscope (Carl Zeiss Surgical, Inc., NY). A volume (0.3 µl) of solution containing fluorescein dye and either nanoparticles, saline (vehicle), or naked DNA was delivered into the subretinal space, usually in the superior temporal quadrant. Since the retina is not fully developed at this age, some injected material is likely released into the vitreous, although the site of injection is subretinal. After injection, the needle was left in place for 3–5 seconds to allow full treatment delivery before being withdrawn gently. Successful delivery of material was confirmed by observation of subretinal yellow-green fluorescence at the time of injection. The cut eyelid was returned to its original position and the surface of the eye was gently blotted with a Kimwipe. Animals were warmed on a temperature-controlled (37°C) bed until fully awake. All nanoparticles and uncompacted plasmid DNA (naked DNA) were used at the same concentration (3.06 µg/µl), selected based on data from our previous study [13]. Because the compaction process relies on the presence of DNA, there is no “empty” nanoparticle, so controls were limited to saline and naked DNA carrying the same therapeutic vector as the nanoparticles. If material delivery could not be confirmed, or if microphthalmia or signs of intraocular infection were observed, the injection was considered unsuccessful and the animal was removed from the study (121/432∼28%). Mice were maintained in the breeding colony under cyclic light (14-hour light/10-hour dark) conditions; cage illumination was approximately 7 foot-candles during the light cycle.

### RNA isolation and qRT-PCR

Both injected and uninjected whole eyes were collected at PI-2, 7, 14, 21, 30, and 120 days for analysis of mRNA levels. qRT-PCR was performed with a MyIQ single-color qRT-PCR machine (Bio-Rad). Mice were euthanized, eyes were enucleated and homogenized and total RNA was extracted using TRIzol (Invitrogen Inc. Carlsbad, CA) as described previously [13]. Subsequently, DNase treatment was performed with RNase-free DNase (Promega Inc.) to remove both genomic DNA and any remaining nanoparticle DNA. cDNA synthesis by reverse transcription was performed and 20 ng of cDNA from each sample was used for qPCR. *Rds* primer sequences were reported previously [13]. Melting curve analysis and agarose gel electrophoresis were performed at the end of the reaction to ensure that the PCR products were specific and of appropriate size. All experimental mRNA levels were quantified against the housekeeping gene hypoxanthine phosphoribosyltransferase (HPRT) as described previously [13]. Relative expression levels were calculated by the 2^−ΔcT^ method [48]. At least three injected and three uninjected eyes from each treatment group at each of the scheduled time points were analyzed. Two independent qPCR experiments for each set of samples were performed and individual samples were run in triplicate. For each sample, the six values (three from two separate reactions) were averaged to get an expression value. The S.E.M. shown in Figure 1 represents the variation from sample to sample (i.e. inter-mouse variation). *Rds* primers amplify from transferred *Rds* and WT *Rds*, but not from the *Rds* mutant allele. To confirm that *Rds* levels were not artificially altered by the presence of undigested nanoparticle, control reactions amplifying from the IRBP or CBA promoter regions were performed and no product was detected. As an additional control, a subset of samples was analyzed with *Rds* primers, but without the addition of reverse transcriptase and no amplification was detected.

### Antibodies

Antibodies were procured and used for immunohistochemistry (IHC) and Western blot (WB) analysis as follows: mAB 3B6 (a kind gift from Dr. R.S. Molday, University of British Columbia, Vancouver, BC, Canada, IHC-1∶100); RDS-CT recognizing both endogenous Rds and, to a lesser extent, NMP (generated in-house, IHC-1∶100, WB-1∶1000); anti-S-opsin, recognizing short-wavelength mouse cone opsin (generated in-house, IHC-1∶100, WB-1∶1000); mAb 1D4, recognizing rhodopsin (a generous gift from Dr. R.S. Molday, WB-1∶5000); Rom-1 (generated in-house, WB-1∶1000); and β-actin-HRP (Sigma/Aldrich, WB-1∶5000).

### Immunohistochemistry

Whole eyes were enucleated and fixed with phosphate-buffered saline containing 4% paraformaldehyde at 4°C overnight. With the exception of PI-2 eyes, the cornea and lens were removed and the eye was returned to fixative for an additional two hours. The eyes were cryoprotected by serial immersion in 15% and 30% (w/v) sucrose solutions for at least two hours each. Individual eyes were embedded in M1 embedding medium (Thermo Electron Corporation, PA) and frozen on dry ice; frozen sections (10 µm thickness) aligned with the vertical meridian were cut with a cryostat (Leica) and collected on precleaned Superfrost-plus® microscope slides (Fisher Scientific). The entire eye was sectioned, and every sixth section was collected, enabling examination of gene expression throughout the retina. For immunohistochemistry, all steps were carried out at room temperature as described previously [13], [47]. Staining controls included eyes from age-matched NMP transgenic and WT mice, and slides on which primary or secondary antibodies were omitted. Observation and imaging were performed using an epifluorescent microscope (AxiophotZeiss Ltd., Germany) and a spinning disk confocal microscope (BX62 Olympus, Japan).

### Protein detection by western blot

Western analysis was performed as reported previously [24], [35], [49]. Dissected individual retinas were homogenized on ice and solubilized (50 mM Tris, pH 7.8, 100 mM NaCl, 5 mM EDTA, 0.05% SDS, 1 mM PMSF, 1% TX-100, 2.5% (v/v) glycerol) for one hour at 4°C, then processed for SDS-PAGE and subsequent Western blotting using 10–50 µg protein per lane (as detected by Bradford assay; Bio-Rad). Blots were imaged using a Kodak Image Station 4000R with Kodak MI software.

### Electroretinography

Full-field electroretinography was performed as previously reported [40] and analysis of the obtained electroretinograms (ERGs) was performed as described in detail elsewhere [24], [40] to quantitatively assess rod- and cone-mediated visual function. Both scotopic (rod) and photopic (cone) ERGs were recorded at PI-30, PI-60, and PI-120 on animals from all treatment groups and controls.

### Histology and electron microscopy

Enucleated eyes were fixed, sectioned, and processed as described previously [50]. Semithin sections collected through the vertical meridian were stained with 1% (w/v) toluidine blue in 1% (w/v) sodium borate, coverslipped, and viewed with an Olympus BH-2 microscope under 63×. Digital images were captured using a Nikon DXM1200 digital camera. Ultrathin sections were stained with 2% uranyl acetate and lead citrate and imaged using a JEOL 100CX electron microscope (80 KeV).

### Statistical analyses

For qRT-PCR data, values are expressed as ratios (to uninjected eye) of mean relative expression (±S.E.M.). Since ratios have an inherently skewed (non-Gaussian) distribution, data were log-transformed before undergoing one-way ANOVA. For ERG data, nanoparticle-injected groups were compared with naked DNA-injected groups. All groups passed the Kolmogorov-Smirnov test for normality and *P*-values are from two-tailed, unpaired Student's t-tests. In cases where unequal variance was found (per an F-test), Welch's correction was applied (http://web.uccs.edu/lbecker/SPSS/ttest.htm). Means and standard errors are reported in Table 1 as indicated. To determine whether there was any significant change in ERG amplitude over time, two-way ANOVA with Bonferroni's post-hoc test was used to compare treatment groups at different ages. To determine whether naked DNA caused any effect on function, one-way ANOVA with Bonferroni's post-hoc test was used to compare CBA-NMP naked DNA, IRBP-NMP naked DNA, and saline injected groups.

## Supporting Information

Figure S1 Transmission electron microscopy of compacted DNA nanoparticles. During compaction of the NMP expression plasmid, the presence of acetate as the lysine counterion produces rod shaped particles with a minor diameter ∼8 nm. Scale bar, 100 nM. EMs prepared as per Fink et al. (Fink TL, Klepcyk PJ, Oette SM, Gedeon CR, Hyatt SL, et al. (2006) Plasmid size up to 20 kbp does not limit effective in vivo lung gene transfer using compacted DNA nanoparticles. Gene Ther 13: 1048–1051.).
(5.87 MB TIF)

Figure S2 Immunofluorescence demonstrating the specificity of the 3B6 antibody for transgenic (NMP) RDS. Sections from WT or NMP transgenic retinas were stained with RDS-CT (which recognizes both transgenic and endogenous RDS) and mAB 3B6 (which recognizes only transgenic RDS).
(9.47 MB TIF)

Figure S3 Transferred NMP is not expressed in saline injected eyes. Frozen retinal sections from eyes collected at multiple ages (PI-2 to PI-30) were immunostained for NMP (mAB 3B6, green) and total RDS (RDS-CT, red) with a nuclear counterstain (DAPI, blue). No NMP is detected in saline-injected control eyes, but native RDS is detected only with RDS-CT antibody beginning at PI-2 (P7), consistent with normal ocular development. Scale bars, 20 µm. N = 3–5 mice per group.
(9.92 MB TIF)

Figure S4 Morphometric analysis of nanoparticle injected eyes. Rows of nuclei (top row) and OS thickness (bottom row) were measured in 3–5 eyes per group. The average of 10 uninjected control eyes is shown by the gray dashed line, ±standard deviation (shaded in gray). Black lines represent results from two individual nanoparticle injected animals. N, nasal side; T, temporal side. At PI-30 no substantial changes in the number of ONL rows are detected. CBA-NMP and IRBP-NMP injected animals show some increase in OS layer thickness near the injection site. At PI-120 CBA-NMP injection has no effect on retinal morphometry, while IRBP-NMP mediates moderate increases in both OS layer thickness and the number of rows of ONL nuclei.
(7.06 MB TIF)

Figure S5 IRBP promoter expression in rods and cones. Eyes were collected and sectioned from transgenic mice expressing NMP under the control of the IRBP promoter. 3B6 specifically recognizes transgenic NMP (not endogenous RDS) and S-opsin labels blue cone photoreceptor outer segments. Note the enhanced 3B6 immunoreactivity in cones (compared to rods) suggesting that the IRBP promoter drives more gene expression in cones than in rods. ROS, rod outer segments, COS, cone outer segments, IS, inner segments. Scale bar, 10 µm.
(3.45 MB TIF)

Text S1 Supplemental Methods
(0.03 MB DOC)
