# Supplementary figures and images for: A Partial Structural and Functional Rescue of a Retinitis Pigmentosa Model with Compacted DNA Nanoparticles
Source: PLoS One. 2009 Apr 24;4(4):e5290. doi: 10.1371/journal.pone.0005290 (PMC2669177; doi:10.1371/journal.pone.0005290)

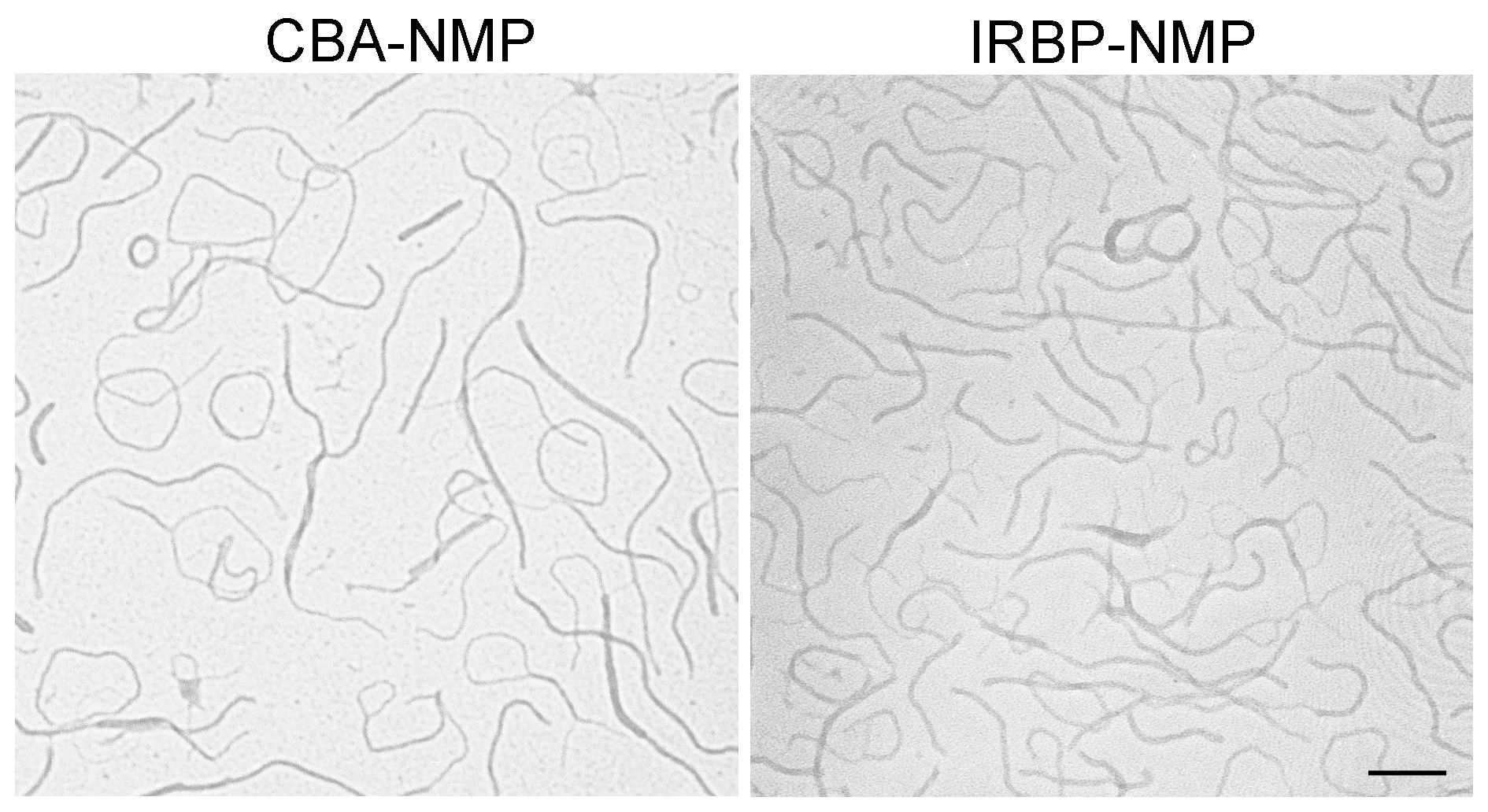

Supplement: Figure S1 — Transmission electron microscopy of compacted DNA nanoparticles. During compaction of the NMP expression plasmid, the presence of acetate as the lysine counterion produces rod shaped particles with a minor diameter ∼8 nm. Scale bar, 100 nM. EMs prepared as per Fink et al. (Fink TL, Klepcyk PJ, Oette SM, Gedeon CR, Hyatt SL, et al. (2006) Plasmid size up to 20 kbp does not limit effective in vivo lung gene transfer using compacted DNA nanoparticles. Gene Ther 13: 1048–1051.). (5.87 MB TIF) [file pone.0005290.s001.tif]

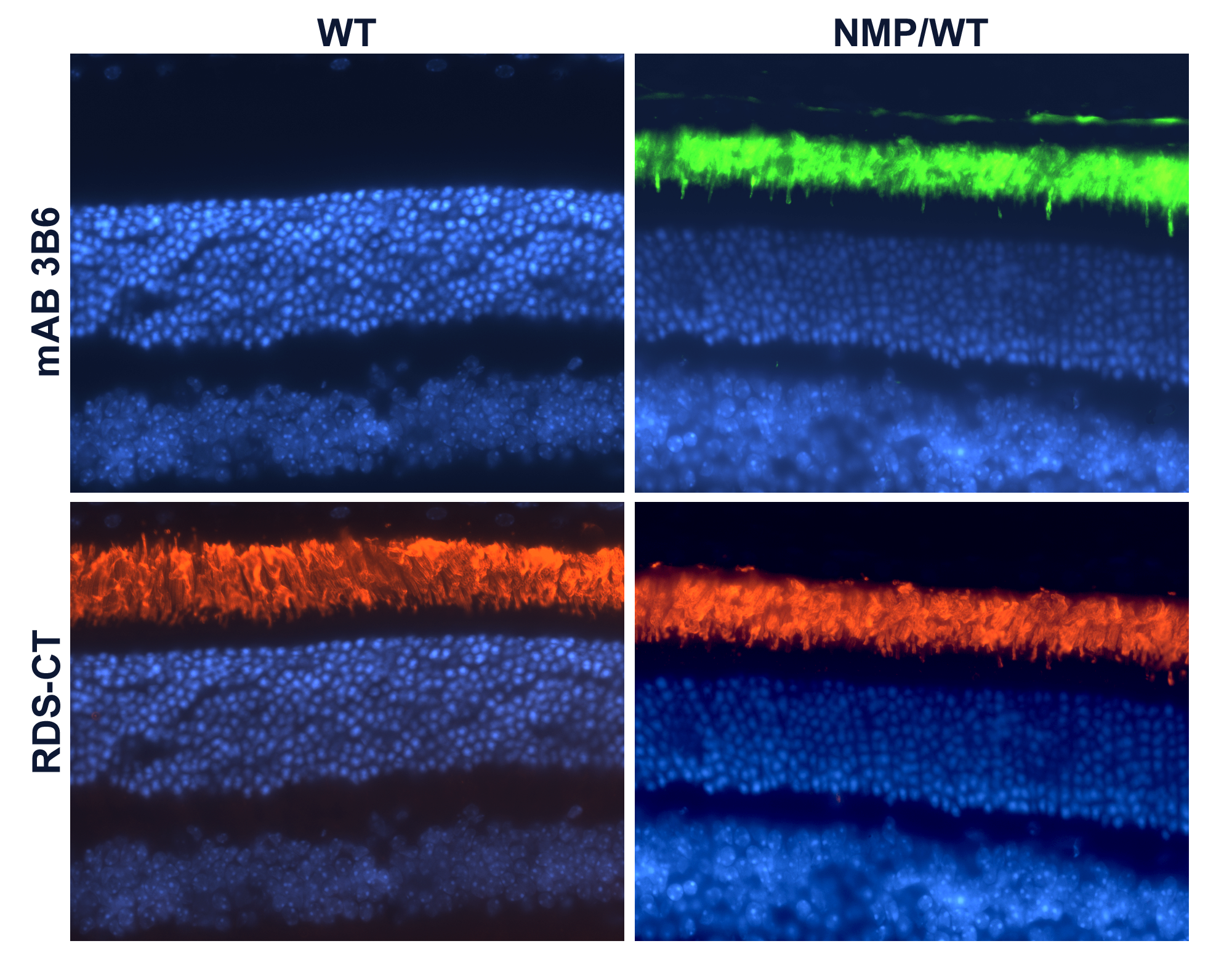

Supplement: Figure S2 — Immunofluorescence demonstrating the specificity of the 3B6 antibody for transgenic (NMP) RDS. Sections from WT or NMP transgenic retinas were stained with RDS-CT (which recognizes both transgenic and endogenous RDS) and mAB 3B6 (which recognizes only transgenic RDS). (9.47 MB TIF) [file pone.0005290.s002.tif]

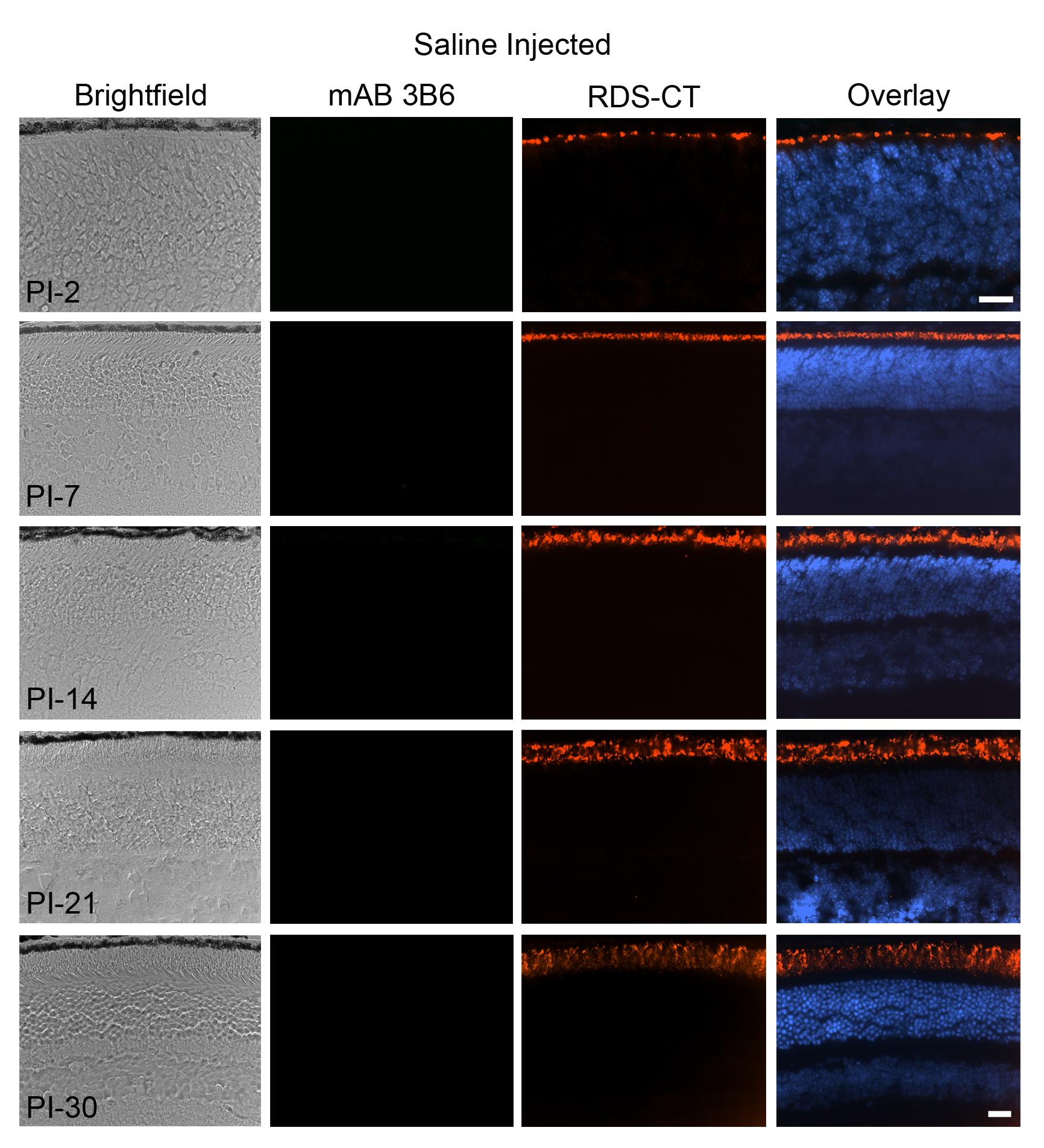

Supplement: Figure S3 — Transferred NMP is not expressed in saline injected eyes. Frozen retinal sections from eyes collected at multiple ages (PI-2 to PI-30) were immunostained for NMP (mAB 3B6, green) and total RDS (RDS-CT, red) with a nuclear counterstain (DAPI, blue). No NMP is detected in saline-injected control eyes, but native RDS is detected only with RDS-CT antibody beginning at PI-2 (P7), consistent with normal ocular development. Scale bars, 20 µm. N = 3–5 mice per group. (9.92 MB TIF) [file pone.0005290.s003.tif]

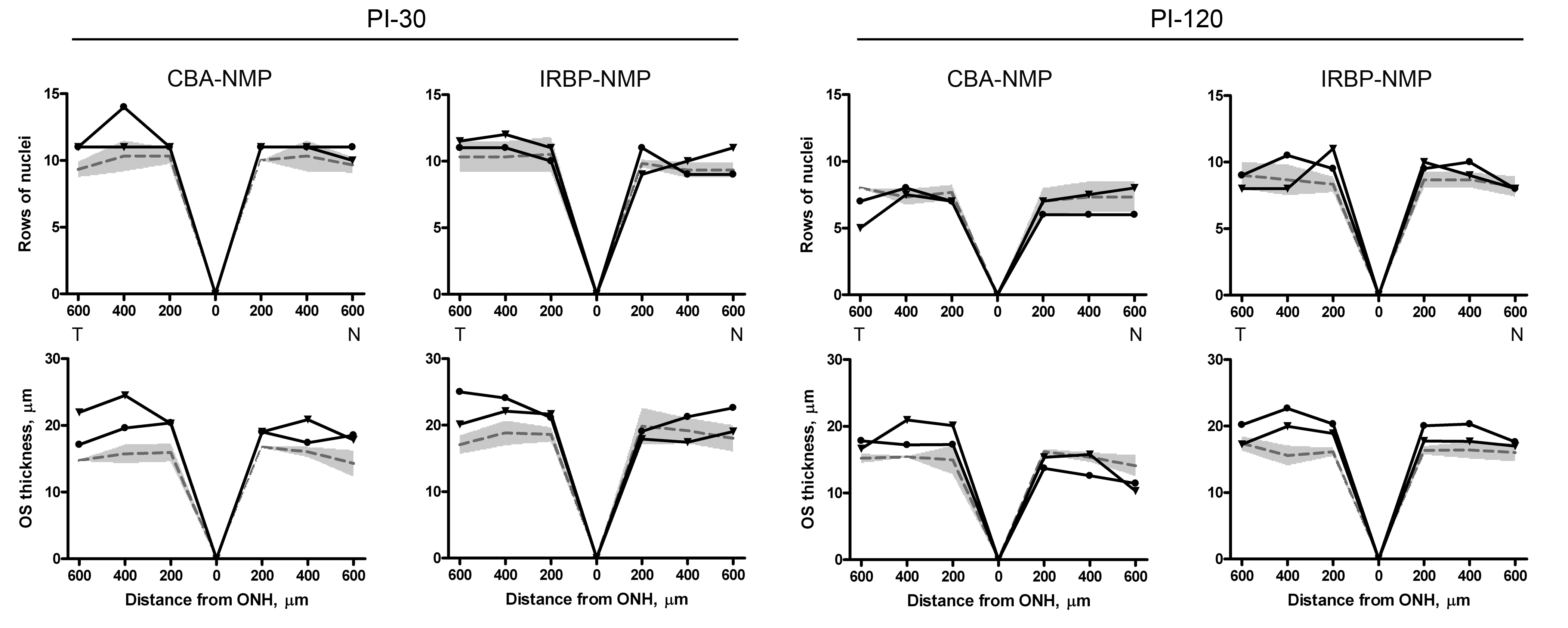

Supplement: Figure S4 — Morphometric analysis of nanoparticle injected eyes. Rows of nuclei (top row) and OS thickness (bottom row) were measured in 3–5 eyes per group. The average of 10 uninjected control eyes is shown by the gray dashed line, ±standard deviation (shaded in gray). Black lines represent results from two individual nanoparticle injected animals. N, nasal side; T, temporal side. At PI-30 no substantial changes in the number of ONL rows are detected. CBA-NMP and IRBP-NMP injected animals show some increase in OS layer thickness near the injection site. At PI-120 CBA-NMP injection has no effect on retinal morphometry, while IRBP-NMP mediates moderate increases in both OS layer thickness and the number of rows of ONL nuclei. (7.06 MB TIF) [file pone.0005290.s004.tif]

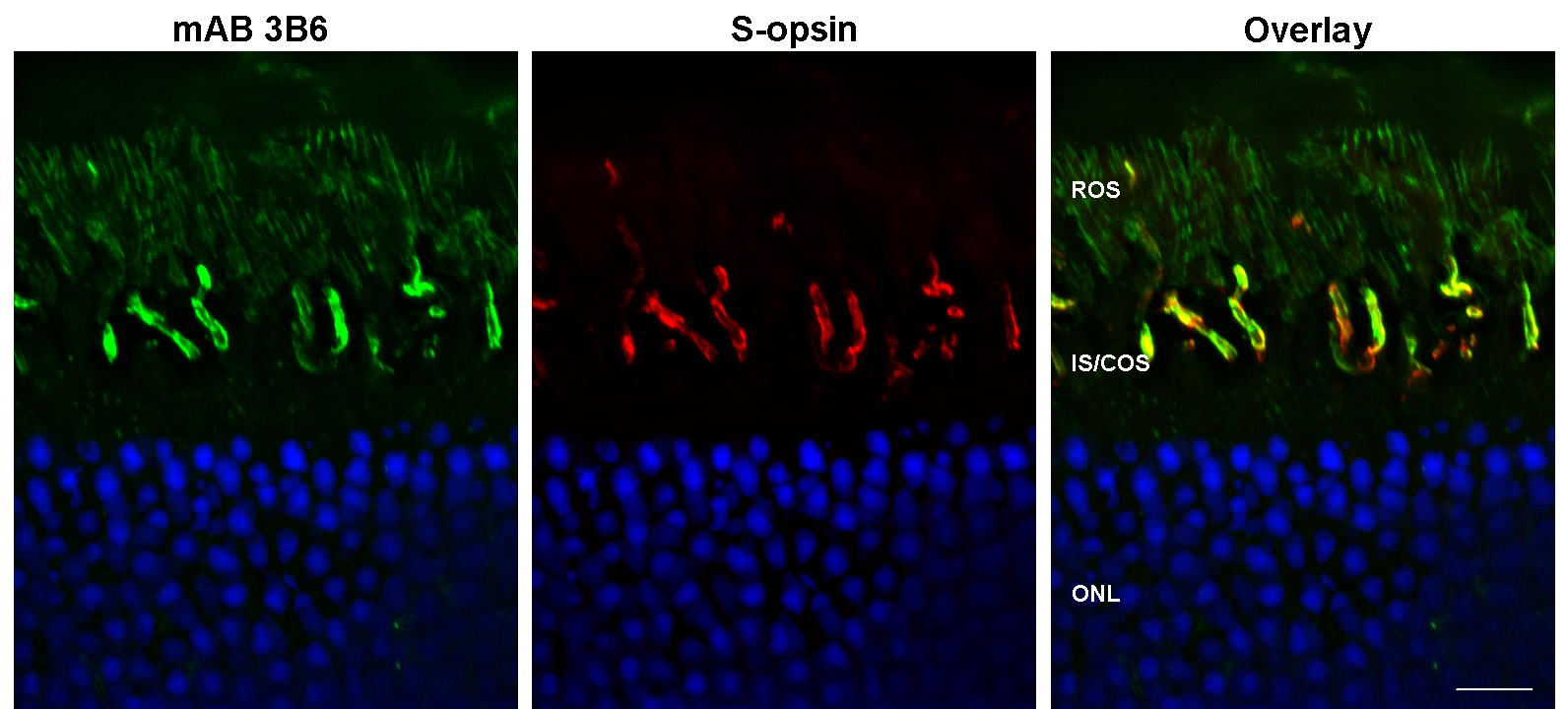

Supplement: Figure S5 — IRBP promoter expression in rods and cones. Eyes were collected and sectioned from transgenic mice expressing NMP under the control of the IRBP promoter. 3B6 specifically recognizes transgenic NMP (not endogenous RDS) and S-opsin labels blue cone photoreceptor outer segments. Note the enhanced 3B6 immunoreactivity in cones (compared to rods) suggesting that the IRBP promoter drives more gene expression in cones than in rods. ROS, rod outer segments, COS, cone outer segments, IS, inner segments. Scale bar, 10 µm. (3.45 MB TIF) [file pone.0005290.s005.tif]
